# Estimation of age and sex-specific Glomerular Filtration Rate and its association with mortality and atherosclerotic cardiovascular outcomes in the Abu Dhabi population; A Retrospective Cohort Study

**DOI:** 10.1007/s40620-025-02347-w

**Published:** 2025-08-05

**Authors:** Latifa Mohammad Baynouna AlKetbi, Yousef Boobes, Bachar Afandi, Hamda Aleissaee, Noura AlShamsi, Mohammed AlMansoori, Ahmed Hemaid, Muna Jalal AlDobaee, Noura AlAlawi, Rudina Mubarak AlKetbi, Toqa Fahmawee, Basil AlHashaikeh, AlYazia AlAzeezi, Fatima Shuaib, Jawaher Alnuaimi, Esraa Mahmoud, Nayla AlAhbabi, Nico Nagelkerke

**Affiliations:** 1 Ambulatory Healthcare Services, SEHA, Abu Dhabi, United Arab Emirates; 2 College of Medicine, United Arab Emirates University, Al Ain, United Arab Emirates; 3 Seha Kidney Care, Tawam Hospital, Abu Dhabi, United Arab Emirates; 4 Tawam Hospital, SEHA, Al Ain, United Arab Emirates

**Keywords:** Risk factors, Chronic kidney disease, Renal hyperfiltration, Secular trends, eGFR

## Abstract

**Background:**

The impact of reduced kidney function quantified by estimated Glomerular Filtration Rate (eGFR) on various adverse clinical outcomes has been extensively studied. This study aims to estimate the age and sex-specific eGFR in the Abu Dhabi population and its association with adverse outcomes.

**Methods:**

This is a retrospective cohort study conducted in 8699 participants enrolled in a national cardiovascular disease screening program from 2011 to 2013. A reference eGFR percentile was estimated from healthy cohort members who had no comorbidities. The  LMS (Lambda, Mu, and Sigma) method was used to determine these percentiles. The cohort was reassessed in 2023 for mortality and cardiovascular outcomes.

**Results:**

The reference percentiles of normal eGFR values showed a marked decrease with age, with small sex differences in the reference percentile distribution. Subjects in the two categories within the higher eGFR threshold range, the 95th and 97th percentiles were older, had a significantly higher prevalence of diabetes, were more frequently smokers, and had higher body mass index, higher HbA1c, higher HDL, lower vitamin D, and were more likely to be males, with higher physical activity and a lower prevalence of coronary heart disease. Older age, female sex, history of atherosclerotic cardiovascular disease, history of hypertension, being treated for hypertension, lower diastolic blood pressure, higher systolic blood pressure, lower HDL, higher HbA1c, and higher vitamin D were significantly associated with lower eGFR percentiles.

**Conclusions:**

These ethnicity-specific eGFR reference values are valuable for the early identification of patients with chronic kidney disease, allowing for early qualification for beneficial preventive medication, evaluations, and nephrologist referrals. A prognostic definition of the higher eGFR threshold, as renal hyperfiltration, is suggested since the 97th percentile had a significantly higher incidence of atherosclerotic cardiovascular disease.

**Graphical abstract:**

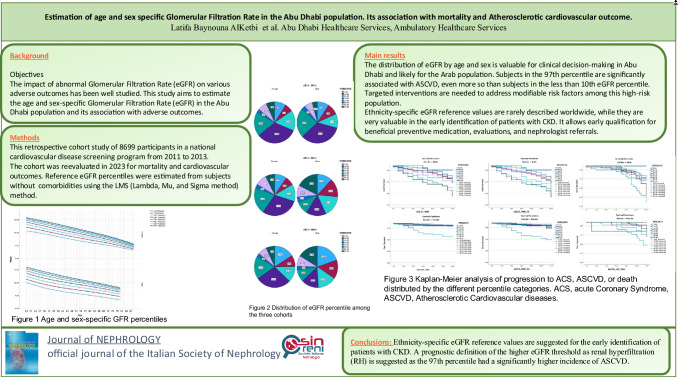

**Supplementary Information:**

The online version contains supplementary material available at 10.1007/s40620-025-02347-w.

## Introduction

Renal impairment adversely impacts patients' health beyond just kidney outcomes, and may result in kidney failure. It is strongly linked to mortality, atherosclerotic cardiovascular disease (ASCVD), and all-cause and cardiovascular death [1, 2]. Renal impairment is classified based on the KDIGO classification of Chronic Kidney Disease (CKD) by cause, glomerular filtration rate (GFR) (G1–G5), and albuminuria (A1–A3) [3]. Nevertheless, this classification is challenged by the generalizability of cutoff points, which do not account for adjustments for age and ethnicity, and can impact management decisions for such diverse populations.

Furthermore, definitions of renal impairment are based on proteinuria or lower estimated GFR (eGFR) thresholds, while higher eGFR thresholds, albeit correlated with adverse outcomes, such as kidney damage and accelerated CKD progression [4], are not classified as a disease state or as a risk factor, and there are no established upper cutoff points between normal and higher eGFR disease states [5]. This is despite evidence of the effectiveness of risk factor management and the availability of renin–angiotensin–aldosterone system (RAAS) blockers and sodium-glucose cotransporter-2 inhibitors (SGLT2i) [6, 7, #182366]. Therefore, defining “normal” ranges is a crucial research area that could influence patient outcomes.

Another important area of research concerns estimating the GFR of different ethnicities. eGFR is affected by body composition and food preferences, among other factors, and applying the Classification of CKD to different ethnicities requires more research to better understand which adjustments are needed. This is important in the West, the region where the current normal values were derived, and in the rest of the world. Currently, the consensus is that incorporating race in GFR estimation still faces challenges, and adjusting for ethnicity could lead to an inaccurate estimation of GFR due to genetic diversity within racial groups [8]. No studies in the UAE or the Arab region have estimated the GFR or determined cutoff points based on adverse events. This study aimed to determine the UAE's eGFR (ab)normal percentiles, associated risk factors, and their relation to adverse outcomes. This research contributes to the global understanding of variations in estimated GFR levels and highlights the need for routine GFR assessments and targeted interventions to prevent CKD and its complications.

## Methods

### Study design

This study is divided into two parts; the first is a retrospective cohort study estimating GFR and its percentiles from 8699 healthy participants involved in a national screening program from 2011 to 2013. At the end of the follow-up period in 2023, the eGFR percentiles were studied in relation to cardiovascular and mortality outcomes for all participants.

The second part of the study uses a cross-sectional design to assess secular trends in estimated GFR percentiles by age and sex. The prevalence of eGFR percentiles was evaluated in two cohorts from the same national screening program at two time points: 2016–17 and 2023–24.

### Setting

In the first part, cohort recruitment was carried out between 2011 and 2013 as part of the cardiovascular prevention program, Weqaya (“prevention”), offered for free by the government to all nationals within the primary healthcare centers in the Emirate of Abu Dhabi, United Arab Emirates (UAE). The participants were UAE nationals living in the Emirate of Abu Dhabi. This cohort was described in a previous publication, the Abu Dhabi Risk Study (ADRS), and subjects were randomly recruited from the community [9]. The dataset was used to construct eGFR percentiles for the healthy population and study outcomes related to baseline characteristics from 2011 to 2013. In 2023, 8,699 subjects from the ADRS cohort were reassessed, with a mean follow-up of 9.4 years. The inclusion criteria for the study were UAE nationals who took part in Weqaya screenings and had a complete dataset of variables at baseline.

Baseline Weqaya assessments took place from 2011 to 2013, and in 2023, doctors and nurses conducted chart reviews to assess various outcomes of interest, such as cardiovascular events, diabetes, and other conditions. Detailed descriptions of the ADRS are available in earlier publications [10]. Recorded variables included most conventional risk factors such as age, sex, Body Mass Index (BMI), HbA1c, vitamin D levels, creatinine, Systolic Blood Pressure (SBP), Diastolic Blood Pressure (DBP), lipid profile (including total cholesterol and HDL), and history of CKD, diabetes, hypertension, and smoking. The eGFR was calculated using the 2021 CKD Epidemiology Collaboration (CKD-EPI) creatinine [11].

The LMS method (Lambda, Mu, and Sigma method) was used to derive the percentiles of eGFR from this dataset to determine the degree to which a person from Abu Dhabi has normal kidney function comparable to the normal Abu Dhabi population. Several mathematical methods have been described for obtaining percentile curves. Nevertheless, the LMS method is one of the most well-known and was first developed by Cole [12]. This method offers a means to produce normalized and smoothed centile curves [13]. The method has also been used to derive centile curves for a range of growth parameters in children, including those established by the World Health Organization (WHO).

**Fig. 1:**
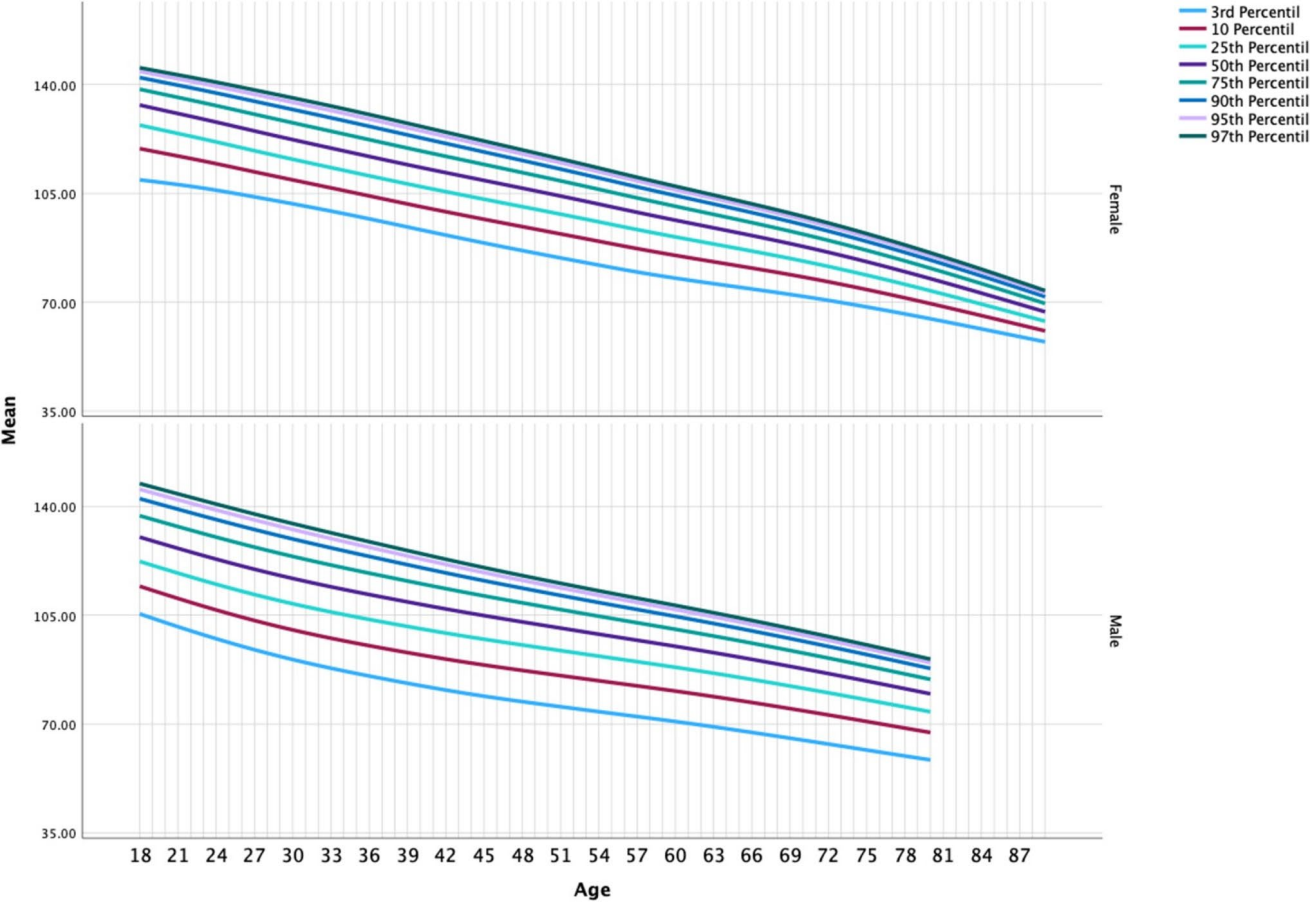
Age and sex-specific GFR percentiles

Only healthy individuals in the ADRS cohort without known comorbidities were included to determine normal percentiles. Excluded subjects had diabetes, hypertension, cancer, heart disease, or stroke. A total of 5113 participants from the whole cohort, 2586 females and 2527 males, were used to construct the percentiles shown in Appendix 1, Fig. 1. In the outcome prediction analysis, these percentiles were applied to all 8699 ADRS cohort participants to assess their eGFR percentiles as a risk factor.

The outcomes studied were death, coronary heart disease, atherosclerotic cardiovascular diseases, and either coronary heart disease or stroke. The relationship between eGFR percentiles and these outcomes was studied. While CKD diagnosis is clearly defined [3], hyperfiltration is not. Two definitions were studied in relation to the occurrence of adverse outcomes: an eGFR above the 95th percentile [14] and the National Kidney Foundation (NKF) criteria, with a GFR of 120 mL/min/1.73 m^2^ or more regardless of age or sex [15].

In part two, to assess changes in prevalence, different participants in the Abu Dhabi preventive screening program in the years 2016–2017 and 2023–2024, a voluntary continuation of Weqaya screening, were assessed for changes in the prevalence of eGFR percentiles over the last decade. The 2016–2017 sample included 2554 subjects, and 2023–2024 included 10,895 subjects. Both datasets were cross-sectional and included an electronic medical record extract report containing age, sex, BMI, and eGFR.

### Statistical analysis

The ADRS data were analyzed using the SPSS analysis program version 29. Descriptive statistics were used and presented as mean ± standard deviation (SD) for continuous variables and as percentages for categorical variables. Ordinal proportional odds models were used to explore the odds ratios (ORs) of associations between eGFR percentiles and other baseline risk factors and their 95% confidence interval (CIs) The all-cause mortality risks and atherosclerotic cardiovascular disease events were estimated using multivariate Cox regression analysis, adjusting for potentially confounding variables such as age, sex, BMI, smoking status, HbA1c, history of diabetes mellitus (DM), history of hypertension, vitamin D level, systolic and diastolic blood pressure, HDL, and total cholesterol. A significance level of *p* < 0.05 was maintained throughout. No imputation was performed for missing data, as it was very minimal.

## Results

At baseline, subjects had a mean age of 38.8 years, BMI of 28.8, and eGFR of 111.3 mL/min/1.73 m^2^. Among males, 16.3% reported current smoking, but smoking was rare among females. Diabetes prevalence was 22.2%, and hypertension prevalence was 19.3% (Appendix 2).

### Estimated GFR percentile prevalence

In both sexes, a marked decrease of eGFR was observed with age (Appendix 1, Appendix 3). There was also a sex difference in the percentile distribution, with females losing GFR faster than males, clearly invalidating the idea of having a fixed age-sex independent cut-off value, notably 120 ml/min/1.73 m^2^, for hyperfiltration. The median of 121.9 ml/min/1.73 m^2^ for males and 126.97 ml/min/1.73 m^2^ for females at the age of 25 decreased to 96.38 ml/min/1.73 m^2^ and 95 ml/min/1.73 m^2^ at the age of 60 years in males and females, respectively.

**Fig. 2:**
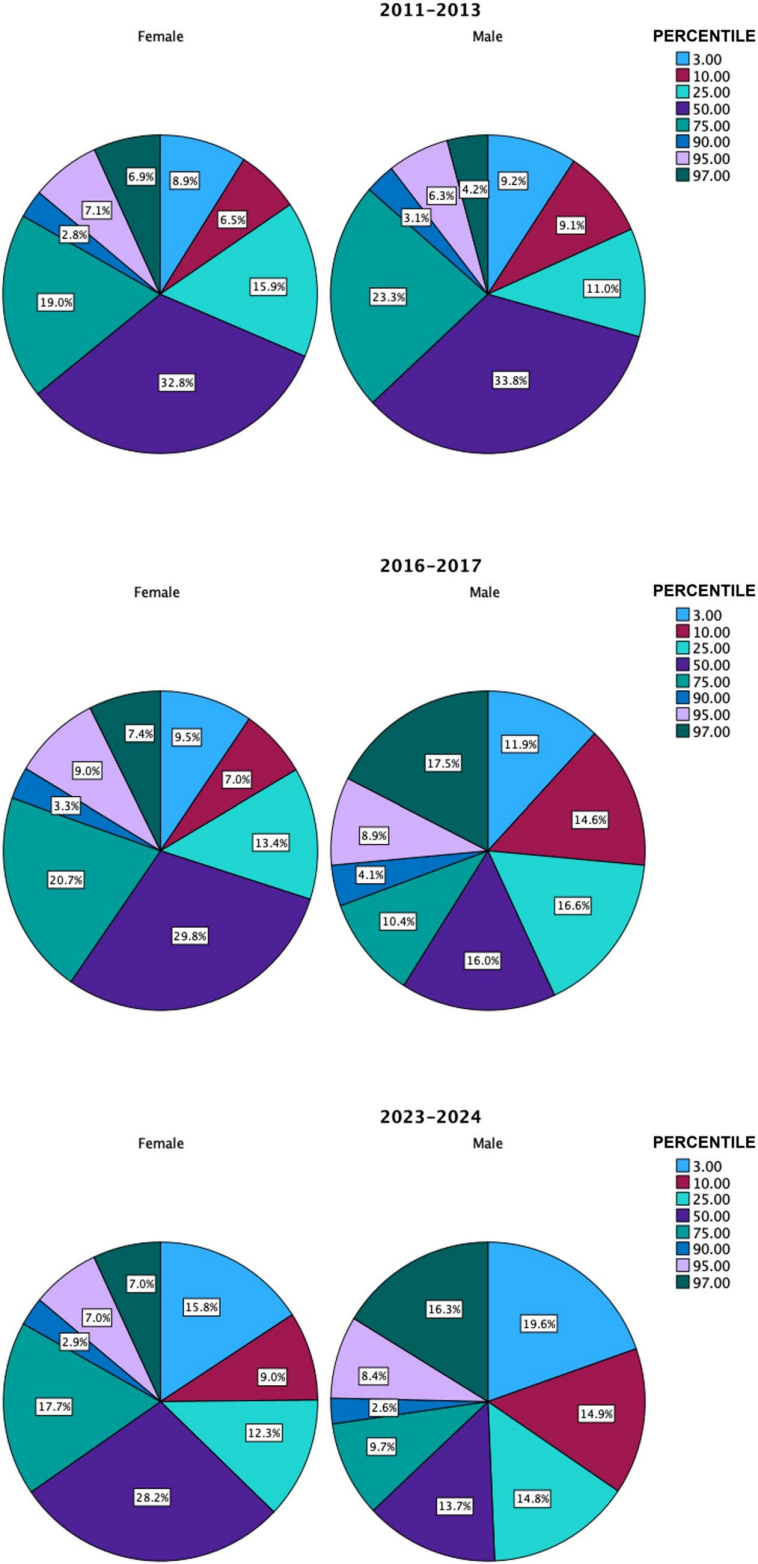
Distribution of eGFR percentile among the three cohorts

Classifying the populations of all cohorts based on eGFR into the established percentiles revealed that 33.8% of males and 32.8% of females fall below the 50th percentile. The distribution of those over the 97th percentile also differed between males and females. It was 4.2% among males, compared to 6.9% for females. Later, in the 2016–2017 cohort, the percentage in excess over expected percentiles increased. There were 17.5% of males exceeding the 97th percentile compared to 7.4% of females. The 2023–2024 percentiles showed a similar distribution to 2016–2017, as shown in Appendix 3, with a 16.3% prevalence of males having an eGFR above the 97th percentile and 7% among females. If we consider hyperfiltration values of eGFR above the 95th percentile for age and sex, the 2011–2013 prevalence nearly doubled among the total population, from 12.2% at baseline to 21.5% in the 2016–2017 and 19.4% in the 2023–2024 cohorts (Fig. 2).

Pregnancy was highly associated with higher eGFR percentiles (OR from univariate logistic regression was 8.6, 95% CI 5.4–14, *p* < 0.001), and therefore, pregnant women were removed from the analysis as pregnancy is a transitional physiological status.

### Associations of eGFR percentiles

Ordinal regression was used to investigate associations of extreme points of eGFR percentiles, i.e., 3rd, 10th, 25th, 95th, and 97th. As shown in Table 1a, lower percentile eGFR percentiles are studied with 3rd percentile as code = 0, 10th–25th code = 1, and 50th–75th as the reference. Table 1b shows higher percentile eGFR, 50th–75th code = 0, 95th code = 1 and 97th as the reference. Significant associations existed between lower percentiles (a) and higher percentiles (b). All variables were collected at baseline in 2011–2013. There was a 4.4% increased risk of a low eGFR for each additional year of age. A history of hypertension at baseline raised the risk by 78%, while coronary heart disease risk increased by 57% for those with lower eGFR percentiles. Additionally, a significant interaction existed between HbA1c levels and diabetes; individuals with diabetes and poor control (higher HbA1c) faced greater risk of being in the lower eGFR percentile.

**Table 1:** Associations of eGFR percentiles with studied risk factors. (a) Lower percentile, eGFR percentiles, 3rd code = 0, 10th–25th code = 1, and 50th–75th as the reference. (b) Higher percentiles. eGFR percentiles, 50th–75th code = 0, 95th code = 1 and 97th as the reference

(a) Ordinal regression of three levels of eGFR percentiles, 3rd, 10th-25th and 50th-75th. The 97th excluded					
		Estimate	*p* value	95% CI	
Threshold	3rd percentile = 0.00	− 2.33	< 0.001	− 3.407	− 1.253
	10th–25th percentile = 1.00]	− 0.748	0.173	− 1.823	0.328
	Reference 50th, 75th and 90th percentiles				
Location	Cholesterol value	− 0.096	< 0.001	− 0.146	− 0.047
	HDL	0.446	< 0.001	0.285	0.606
	Vitamin D	− 0.004	< 0.001	− 0.007	− 0.002
	Interaction of age and diabetes	− 0.013	0.014	− 0.024	− 0.003
	Age	0.044	< 0.001	0.025	0.063
	Age squared	0	< 0.001	− 0.001	0
	SBP	− 0.006	0.009	− 0.011	− 0.002
	DBP	0.005	0.084	− 1.00E− 03	0.012
	Interaction of HbA1c and diabetes	− 0.067	0.051	− 0.134	0
	DM before screening = 0.00	− 1.208	0.002	− 1.956	− 0.46
	DM before screening = 1.00	0a			
	ASCVD before = 0.00	0.429	< 0.001	0.2	0.658
	ASCVD before = 1.00	0a			
	Current smoker = 0.00	− 0.108	0.252	− 0.292	0.077
	Current smoker = 1.00	0a			
	[Sex = 1.00]	− 0.378	< 0.001	− 0.488	− 0.268
	[Sex = 2.00]	0a			
	[On BP treatment = 0]	0.34	0.004	0.107	0.572
	[On BP treatment = 1]	0a			
	[HTN before screening = 0.00]	0.22	0.044	0.006	0.434
	[HTN before screening = 1.00]	0a			

(b) Ordinal regression of three levels of eGFR percentiles, 97th, 95th, and 50th -75th. the 3rd 10th and 25th excluded					
		Estimate	*p* value	95% CI	
Threshold	50th and 75th percentiles = 0 .00	0.995	0.115	− 0.242	2.233
	95th percentile = 1.00	2.322	< 0.001	1.082	3.562
	Reference is 97th percentile				
Location	Cholesterol value	− 0.222	< 0.001	− 0.295	− 0.149
	HbA1c	0.156	< 0.001	0.081	0.231
	HDL	0.643	< 0.001	0.447	0.838
	Vitamin D	− 0.019	< 0.001	− 0.03	− 0.008
	BMI	0.012	0.03	0.001	0.022
	Interaction of age and diabetes	− 0.016	0.018	− 0.029	− 0.003
	Age	− 0.026	0.041	− 0.051	− 0.001
	Square age	0	0.135	− 7.11E−05	0.001
	Interaction of age and vitamin D	0	0.006	9.18E−05	0.001
	[Not active in physical activity = 0]	0.207	0.003	0.072	0.342
	[Active in physical activity = 1]	0a			
	DM before screening = 0.00	− 0.945	0.007	− 1.628	− 0.263
	DM before screening = 1.00	0a			
	ASCVD before = 0.00	0.411	0.045	0.009	0.813
	ASCVD before = 1.00	0a			
	Current smoker = 0.00	− 0.179	0.122	− 0.406	0.048
	Current smoker = 1.00	0a			

*eGFR* estimated glomerular filtration rate, *HbA1c* glycated hemoglobin, *DBP* diastolic blood pressure, *HTN* hypertension, *HDL* high density lipoprotein, *BMI* body mass index, *DM* diabetes mellitus, *BP* blood pressure

There was an interaction between age and diabetes, with older diabetic patients being at higher risk. Surprisingly, diabetes diagnosis correlated with higher percentiles; non-diabetics had a 20.8% higher risk of being in lower percentiles. Smoking did not significantly relate to lower risk percentiles. With regard to associations with higher percentiles, sex and smoking status were not significantly related to higher percentiles (Table 1). For each one-unit increase in HbA1c, there was a 15.6% increase in the risk of being in a higher eGFR percentile. Furthermore, for each one-unit drop of vitamin D, there was a 1.9% increased risk of being in the higher eGFR percentile, i.e., 19% for every ten units; thus, a level of 20 has twice the risk of that of a person with a level of 70. However, higher HDL and lower total cholesterol levels were associated with lower eGFR percentiles (Appendix 4).

Similarly, a one-unit increase in BMI was linked to a 1.2% rise in risk. There was a significant interaction between age and diabetes, with older diabetic patients at higher risk of being in the hyperfiltration range. Additionally, younger subjects with lower vitamin D levels faced a greater risk in that range.

### eGFR percentiles at baseline and outcome assessed in 2023

During the decade of follow-up, 39 females (1% of females) and 94 males (2.5% of males) died, and there were 62 atherosclerotic cardiovascular disease events among females (1.4% of females) and 239 events among males (5.5% of males). Coronary artery diseases occurred in 46 females (1.1% of females) and 204 males (4.7% of males).

**Fig. 3:**
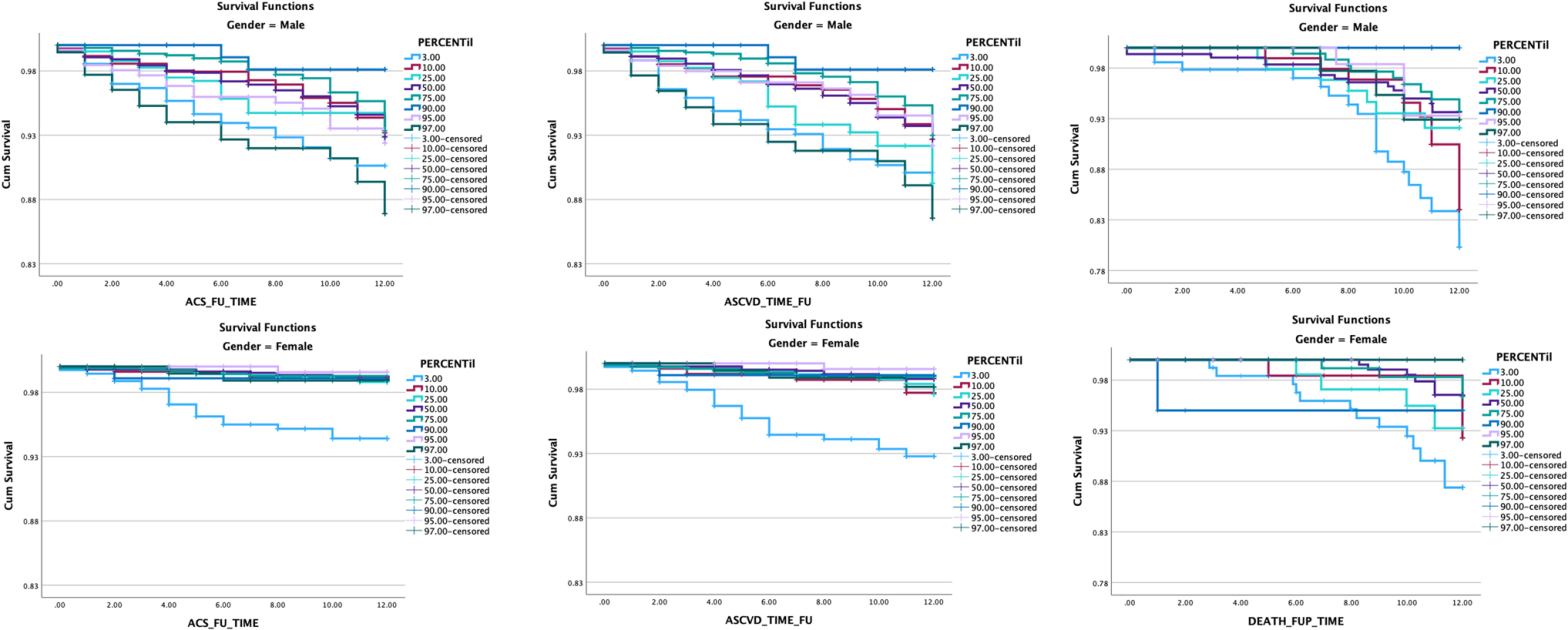
Kaplan–Meier analysis of progression to ACS, ASCVD, or death distributed by the different percentile categories. *ACS* acute coronary syndrome, *ASCVD* atherosclerotic cardiovascular diseases

Using multivariate Cox regression, when renal hyperfiltration was based on the definition of eGFR of more than 120, only weak, non-significant associations were found with death, HR = 1.839 (0.827–4.088) *p* value = 0.135, coronary artery disease, HR 2.058 (0.812–5.216) *p* value 0.128 and atherosclerotic cardiovascular disease, HR = 1.075 (0.465–2.487), *p* = 0.87. However, in the Cox survival analysis, there was a significant relationship between being in the eGFR 97th percentile levels and the incidence of new coronary heart disease and new atherosclerotic cardiovascular disease, HR = 1.65 (1.1–2.6), *p* = 0.024 and HR = 1.53 (1.005–2.4), *p* = 0.048, respectively. This association with the 97th percentile was not significantly associated with mortality. Nevertheless, as shown in Fig. 3, Kaplan–Meier analysis shows a clear survival difference among the different percentile categories, with the 97th percentile having the highest death rate among older males. The higher risk of the 97th percentile is very clear and significant in the case of coronary heart disease and atherosclerotic cardiovascular disease.

## Discussion

This study reports the first eGFR percentiles for an Arab population derived from a community-based population health cohort. The eGFR levels are higher than those reported for Caucasian populations, such as data from Germany [16], while they are closer to the normal values observed in Chinese populations [17]. Similarly, in a 6-year longitudinal population-representative study from Delhi and Chennai, eGFRs in India were higher than those reported in European cohorts [18]. The 3rd percentile for eGFR below 60, i.e., the CKD cutoff, was at age 77 for males and 85 for females. These differences in eGFR across Asian and Caucasian populations require further investigation. Comorbidities and access to healthcare may be contributing factors. Although healthcare access in the UAE is among the best in the world, the prevalence of obesity, diabetes, prediabetes, and other CKD risk factors is very high [9]. Furthermore, differences in body composition between Caucasians and other ethnicities, including Arabs, Indians, and Chinese, may also contribute to the variation. Lower muscle mass, which results in lower creatinine levels, could lead to overestimating true kidney function [19]. A higher eGFR may indicate factors beyond hyperfiltration, such as low serum creatinine from sarcopenia, undernutrition, and diet patterns. These should be assessed in future studies.

The prevalence of higher eGFR thresholds among the Abu Dhabi population is notably higher than that of other regions. In the United States, the highest reported renal hyperfiltration prevalence among 12 to 29-year-old males was 11.8%, above the 95th percentile, particularly among obese individuals [20]. In Italy, among patients receiving specialist diabetes care in a large cohort, the prevalence was 3.4% [21], and in the general population of Korea, it was 2.69% [22]. This study reported a higher prevalence of higher eGFR thresholds than these countries. Higher eGFR level is frequently observed in individuals with obesity, hypertension, and early-stage diabetes, serving as an early warning sign of potential kidney damage and progression to CKD [23]. The high prevalence observed in the Abu Dhabi cohort may be partly attributed to the high rates of obesity, smoking, diabetes, and other related risk factors, underscoring the need for interventions to address these contributing factors.

Renal hyperfiltration possibly presents as higher eGFR thresholds as a result of glomerular and tubular hypertrophy, causing changes in intrarenal hemodynamics, which can lead to glomerulosclerosis and tubulointerstitial injury. This contributes to the development of CKD and cardiovascular disease [24]. These histological changes occur in response to various stimuli such as high-protein intake, hyperglycemia, or insulin resistance, and obesity or metabolic syndrome [3, 5, 15]. As well, a variety of mechanisms have been described in association with different risk factors of renal hyperfiltration. In diabetes, for example, the most common hypothesis is an increase in proximal tubule glucose reabsorption, which involves the upregulation of SGLTs and the growth of the renal tubules. This, in turn, leads the tubuloglomerular feedback to alter the GFR. Furthermore, certain levels of hormones and vasoactive substances are elevated in individuals with diabetes; these substances regulate the contraction and dilation of the pre-glomerular and post-glomerular arterioles, resulting in hyperfiltration by altering vascular resistance [25]. It is worth noting that hypofiltration was not associated with prediabetes or prehypertension, but it did [26] indicate that renal hyperfiltration represents an early change in kidney function. In diabetes, it precedes the onset of albuminuria or the decline in kidney function. On the other hand, vitamin D suppresses the RAAS. The activated form of vitamin D, 1,25(OH)2D, suppresses renin biosynthesis, and vitamin D deficiency stimulates renin production; based on its suppression of the RAAS system in animal models, vitamin D supplementation shows beneficial effects on albuminuria, which suggests that vitamin D supplementation may prevent the progression of kidney disease to kidney failure like other RAAS blockers [27].

Therefore, better management of modifiable risk factors, like smoking cessation, glycemic control, weight management, and ACE inhibitors, reduces the negative health consequences of renal hyperfiltration [21, 28].

Higher HbA1c levels, lower insulin sensitivity, and obesity are linked to renal hyperfiltration, even at prediabetes stages. This study found no significant association between diabetes diagnosis and eGFR values below the 10th percentile. However, the interaction of diabetes with age and HbA1c shows that older, uncontrolled diabetics face higher risks, while younger, well-controlled diabetics have lower risks. In obese patients, eGFR increases with body weight and is associated with albuminuria, indicating early kidney injury [29]. These findings highlight the importance of early detection and intervention, including smoking cessation, in individuals with hyperfiltration to mitigate the risk of progressive kidney damage and improve long-term outcomes [30].

Maeda et al. found that smoking was linked to an elevated risk of glomerular hyperfiltration and proteinuria. In this study, although smoking status was not significantly associated with higher or lower eGFR percentiles, the association between smoking and eGFR percentiles, despite the higher risk reported in other populations [6], warrants further investigation.

The association between hyperfiltration and lower vitamin D levels further suggests the need for early intervention strategies. Vitamin D deficiency has been implicated in the pathogenesis of CKD [31]. Thus, vitamin D supplementation may hold promise in slowing the progression of CKD and preserving kidney function [32]. Emerging evidence suggests vitamin D supplementation may protect kidneys by reducing inflammation and proteinuria, and modulating the renin–angiotensin–aldosterone system.

Further research is needed to clarify the mechanisms underlying the link between hyperfiltration, vitamin D deficiency, and CKD progression, as well as to assess the effectiveness of vitamin D supplementation as an adjunctive therapy in CKD management. A surprising finding that warrants further investigation is the association of lower cholesterol, higher HDL, and lower SBP with hyperfiltration. Elevated HDL-C levels were independently linked to accelerated GFR loss in a middle-aged, nondiabetic population in a large cohort study in Norway. Whether high levels of HDL-C or higher levels of dysfunctional HDL-C contribute to endothelial dysfunction and vascular disease remains unclear. Intrarenal RAAS activation significantly impacts renal hyperfiltration pathogenesis and is usually linked to higher blood pressure. However, our study found that hypertension and elevated blood pressure correlated with a lower risk of renal hyperfiltration. Blood pressure medication did not significantly correlate with renal hyperfiltration, but the potential role of RAAS inhibitors needs further exploration.

Regarding the eGFR, 97th percentile significantly predicted heart disease and atherosclerotic cardiovascular disease, but not mortality, which could be due to the few deaths in this cohort. Nevertheless, this cutoff level suggests a prognostic definition of the higher eGFR threshold, and the 97th percentile level in this cohort shows that there may be ethnic differences regarding which high percentile could represent renal hyperfiltration. In Caucasian populations, the 95th percentile showed high-risk adverse outcomes. Although the observed associations between higher eGFR and adverse health outcomes, including cardiovascular events and mortality, are well-established in nephrology and epidemiology literature [4, 14, 24, 33, 34], there are major gaps in this area. Especially with regard to the definition of pathological cutoff for higher eGFR levels. Utilizing percentiles of eGFR could be the strategy of choice, which, in the era of data-driven healthcare, is very feasible to support healthcare professionals’ and patients’ decisions. Using the 120 ml/min/1.73 m^2^ value of eGFR as suggested by some experts [15] did not perform well in predicting outcomes in this study, and probably, if used, will not account for sex and age, limiting precision in identifying high-risk patients.

Another priority is research investigating the relationship between renal hyperfiltration and atherosclerotic cardiovascular disease to better understand the cogent use of medications. Although it is now suggested that renal hyperfiltration can be a useful surrogate for surveillance of atherosclerotic cardiovascular disease in asymptomatic individuals, the underlying mechanisms driving this association remain largely unknown. It is unclear whether it is a cause-and-effect relationship, and which underlying mechanisms are involved [4, 35]. This is partly due to the lack of consensus on the definitions of renal hyperfiltration or the higher normal eGFR threshold. In the literature, renal hyperfiltration was defined at the 90th to 95th percentiles of eGFR, and at the 99th percentile in adolescents and youth [36], but in this study, the association with adverse events was observed at the 97th percentile. This could reflect the ethnicity effect, variations in atherosclerotic cardiovascular disease epidemiology, or the effect of risk factor management. Therefore, although this study adds to the literature in this area, and specifically for this region of the world, more studies are needed to understand this association.

Reflecting on the implications for patients and healthcare services in the United Arab Emirates, there are national comprehensive preventive screening programs that include kidney function assessment. Local clinical practice guidelines to assess and manage identified risk factors could provide early detection and management for renal hyperfiltration and ameliorate its consequences.

This study has limitations that must be considered when interpreting its findings. Unmeasured confounding factors like socioeconomic status, dietary habits, and health behaviors may influence GFR estimation in the Middle East/Arabian population, just as medication use, evolving comorbidities, or healthcare access can influence disease outcomes over time. Additionally, the lack of dietary sodium and hydration assessments could skew creatinine measurements. Unfortunately, proteinuria is not included in the screening program, and therefore, it was not provided at screening, which limits the understanding of its relation to eGFR and renal hyperfiltration. While the study involved a large cohort, older age subgroups had smaller samples, which may have limited the statistical power to detect significant associations. Moreover, the estimated GFR is derived from serum creatinine without incorporating cystatin C, which could have provided a more accurate assessment of kidney function.

In conclusion, the prevalence of CKD in the UAE is high, making it essential to incorporate all identified risk factors and develop locally relevant GFR estimations for prevention strategies. Ethnicity-specific eGFR reference values are rarely described globally; however, they are vital for the early identification of CKD, enabling timely qualification for beneficial preventive medication, evaluation, and referrals to nephrologists [37].

## Supplementary Information

Below is the link to the electronic supplementary material.

Supplementary file1 (PDF 197 KB)

Supplementary file2 (DOCX 15 KB)

Supplementary file3 (JPG 160 KB)

## Data Availability

Data availability is restricted due to institutional policies.
