# Supplementary material for: Estimation of age and sex-specific Glomerular Filtration Rate and its association with mortality and atherosclerotic cardiovascular outcomes in the Abu Dhabi population; A Retrospective Cohort Study
Source: J Nephrol. 2025 Aug 5;38(7):1957–67. doi: 10.1007/s40620-025-02347-w (PMC12484320; doi:10.1007/s40620-025-02347-w)
Supplement: Supplementary file 1 — Supplementary file1 (PDF 197 KB) [file 40620_2025_2347_MOESM1_ESM.pdf]

Females									
Age	Gender	Percent3	Percent10	Percent25	median	Percent75	Percent90	Percent95	Percent97
14	1.00	110.98	122.62	130.65	137.13	142.04	145.58	147.40	148.49
15	1.00	110.63	121.82	129.72	136.19	141.15	144.75	146.61	147.72
16	1.00	110.24	121.02	128.80	135.26	140.26	143.91	145.81	146.94
17	1.00	109.81	120.21	127.88	134.33	139.38	143.08	145.01	146.17
18	1.00	109.34	119.40	126.96	133.40	138.49	142.25	144.22	145.40
19	1.00	108.86	118.59	126.04	132.48	137.61	141.42	143.42	144.63
20	1.00	108.36	117.80	125.14	131.57	136.73	140.60	142.64	143.86
21	1.00	107.83	117.00	124.25	130.67	135.87	139.78	141.85	143.10
22	1.00	107.28	116.21	123.36	129.77	135.00	138.96	141.06	142.34
23	1.00	106.68	115.39	122.46	128.85	134.11	138.12	140.26	141.55
24	1.00	106.02	114.54	121.54	127.92	133.21	137.27	139.43	140.75
25	1.00	105.33	113.68	120.60	126.97	132.29	136.39	138.59	139.93
26	1.00	104.61	112.79	119.66	126.02	131.37	135.50	137.73	139.09
27	1.00	103.87	111.91	118.72	125.07	130.44	134.61	136.87	138.24
28	1.00	103.14	111.04	117.79	124.12	129.52	133.73	136.01	137.40
29	1.00	102.40	110.18	116.87	123.19	128.60	132.84	135.14	136.55
30	1.00	101.65	109.33	115.96	122.27	127.69	131.96	134.28	135.70
31	1.00	100.88	108.47	115.06	121.36	126.79	131.07	133.41	134.84
32	1.00	100.10	107.62	114.17	120.46	125.89	130.19	132.53	133.97
33	1.00	99.31	106.77	113.29	119.56	124.99	129.29	131.65	133.09
34	1.00	98.49	105.91	112.41	118.66	124.09	128.39	130.75	132.19
35	1.00	97.65	105.04	111.52	117.76	123.18	127.48	129.84	131.28
36	1.00	96.80	104.18	110.64	116.86	122.27	126.56	128.91	130.36
37	1.00	95.94	103.31	109.76	115.97	121.36	125.64	127.98	129.42
38	1.00	95.07	102.45	108.90	115.09	120.46	124.72	127.05	128.48
39	1.00	94.19	101.60	108.04	114.22	119.56	123.80	126.11	127.53
40	1.00	93.31	100.75	107.20	113.35	118.67	122.88	125.17	126.58
41	1.00	92.44	99.91	106.36	112.50	117.79	121.96	124.23	125.63
42	1.00	91.59	99.09	105.55	111.67	116.92	121.05	123.30	124.68
43	1.00	90.74	98.28	104.74	110.83	116.05	120.14	122.37	123.74
44	1.00	89.89	97.48	103.93	110.00	115.18	119.23	121.43	122.78
45	1.00	89.05	96.67	103.12	109.16	114.30	118.32	120.50	121.83
46	1.00	88.22	95.87	102.32	108.33	113.43	117.40	119.56	120.87
47	1.00	87.41	95.09	101.52	107.50	112.56	116.49	118.62	119.92
48	1.00	86.61	94.31	100.73	106.67	111.69	115.58	117.69	118.97
49	1.00	85.82	93.53	99.93	105.84	110.81	114.66	116.75	118.02
50	1.00	85.03	92.74	99.12	105.00	109.92	113.74	115.80	117.06
51	1.00	84.25	91.96	98.31	104.15	109.03	112.81	114.85	116.09
52	1.00	83.48	91.18	97.50	103.29	108.13	111.87	113.89	115.12
53	1.00	82.71	90.39	96.68	102.42	107.22	110.93	112.92	114.14
54	1.00	81.94	89.59	95.85	101.55	106.30	109.97	111.95	113.15
55	1.00	81.18	88.80	95.01	100.67	105.38	109.01	110.96	112.16
56	1.00	80.44	88.02	94.18	99.79	104.45	108.05	109.98	111.16
57	1.00	79.72	87.25	93.36	98.92	103.54	107.10	109.01	110.18
58	1.00	79.03	86.50	92.56	98.06	102.63	106.16	108.05	109.21
59	1.00	78.38	85.78	91.77	97.22	101.74	105.23	107.10	108.25
60	1.00	77.74	85.06	91.00	96.38	100.86	104.31	106.16	107.30
61	1.00	77.13	84.37	90.23	95.56	99.98	103.39	105.23	106.35

62	1.00	76.54	83.69	89.48	94.74	99.11	102.49	104.30	105.41
63	1.00	75.97	83.01	88.72	93.92	98.24	101.58	103.38	104.47
64	1.00	75.40	82.34	87.97	93.10	97.37	100.67	102.45	103.53
65	1.00	74.83	81.66	87.21	92.27	96.49	99.75	101.51	102.58
66	1.00	74.27	80.98	86.45	91.44	95.60	98.82	100.56	101.62
67	1.00	73.69	80.29	85.68	90.59	94.70	97.89	99.60	100.65
68	1.00	73.11	79.59	84.89	89.73	93.79	96.93	98.63	99.67
69	1.00	72.51	78.87	84.08	88.85	92.85	95.95	97.63	98.66
70	1.00	71.89	78.13	83.24	87.94	91.89	94.95	96.61	97.62
71	1.00	71.26	77.36	82.39	87.01	90.90	93.93	95.56	96.57
72	1.00	70.60	76.58	81.51	86.06	89.89	92.87	94.49	95.48
73	1.00	69.91	75.76	80.60	85.07	88.85	91.79	93.39	94.37
74	1.00	69.21	74.93	79.67	84.06	87.78	90.68	92.26	93.23
75	1.00	68.49	74.08	78.72	83.03	86.69	89.55	91.11	92.07
76	1.00	67.76	73.20	77.75	81.98	85.58	88.40	89.94	90.88
77	1.00	67.01	72.32	76.76	80.91	84.45	87.22	88.74	89.67
78	1.00	66.25	71.41	75.75	79.82	83.29	86.03	87.52	88.44
79	1.00	65.47	70.49	74.73	78.71	82.12	84.81	86.28	87.18
80	1.00	64.68	69.55	73.69	77.58	80.93	83.57	85.02	85.91
81	1.00	63.88	68.60	72.63	76.44	79.71	82.31	83.73	84.61
82	1.00	63.07	67.65	71.56	75.28	78.49	81.04	82.44	83.30
83	1.00	62.25	66.68	70.48	74.11	77.25	79.75	81.12	81.97
84	1.00	61.42	65.70	69.40	72.93	76.00	78.44	79.79	80.62
85	1.00	60.59	64.72	68.30	71.74	74.73	77.13	78.45	79.27
86	1.00	59.76	63.74	67.20	70.54	73.46	75.80	77.10	77.90
87	1.00	58.93	62.75	66.10	69.34	72.18	74.47	75.73	76.52
88	1.00	58.10	61.77	64.99	68.13	70.89	73.12	74.36	75.13
89	1.00	57.27	60.78	63.89	66.92	69.61	71.78	72.98	73.73

Males									
Age	Gender	Percent3	Percent10	Percent25	median	Percent75	Percent90	Percent95	Percent97
14	2.00	111.13	119.69	127.40	134.98	141.65	146.99	149.91	151.70
15	2.00	109.69	118.33	126.12	133.76	140.49	145.87	148.81	150.62
16	2.00	108.26	116.99	124.84	132.55	139.33	144.74	147.71	149.53
17	2.00	106.84	115.65	123.57	131.33	138.16	143.61	146.60	148.43
18	2.00	105.43	114.32	122.30	130.12	136.99	142.48	145.48	147.33
19	2.00	104.03	112.99	121.03	128.90	135.82	141.34	144.37	146.22
20	2.00	102.65	111.68	119.77	127.70	134.65	140.21	143.25	145.12
21	2.00	101.30	110.39	118.53	126.50	133.50	139.08	142.13	144.01
22	2.00	99.98	109.13	117.31	125.32	132.35	137.95	141.02	142.91
23	2.00	98.69	107.89	116.12	124.16	131.21	136.84	139.92	141.82
24	2.00	97.44	106.69	114.95	123.02	130.10	135.74	138.83	140.73
25	2.00	96.22	105.52	113.82	121.91	129.01	134.66	137.75	139.65
26	2.00	95.04	104.39	112.72	120.83	127.94	133.59	136.69	138.59
27	2.00	93.90	103.30	111.65	119.78	126.89	132.54	135.64	137.54
28	2.00	92.81	102.25	110.63	118.76	125.87	131.51	134.60	136.50
29	2.00	91.75	101.24	109.64	117.78	124.87	130.50	133.58	135.47
30	2.00	90.74	100.27	108.69	116.82	123.90	129.51	132.58	134.46
31	2.00	89.78	99.34	107.77	115.90	122.96	128.54	131.59	133.46
32	2.00	88.85	98.46	106.89	115.00	122.03	127.59	130.62	132.48
33	2.00	87.96	97.60	106.04	114.13	121.13	126.65	129.66	131.50
34	2.00	87.10	96.78	105.21	113.29	120.25	125.73	128.71	130.53
35	2.00	86.26	95.98	104.42	112.46	119.38	124.81	127.77	129.58
36	2.00	85.45	95.21	103.64	111.65	118.52	123.91	126.84	128.63
37	2.00	84.66	94.45	102.88	110.85	117.67	123.02	125.91	127.68
38	2.00	83.89	93.71	102.13	110.06	116.83	122.12	124.98	126.73
39	2.00	83.12	92.98	101.39	109.29	116.00	121.23	124.06	125.79
40	2.00	82.37	92.27	100.66	108.52	115.17	120.35	123.14	124.85
41	2.00	81.64	91.57	99.95	107.76	114.36	119.48	122.24	123.92
42	2.00	80.93	90.90	99.26	107.02	113.55	118.61	121.34	123.00
43	2.00	80.25	90.24	98.58	106.29	112.76	117.76	120.45	122.09
44	2.00	79.59	89.61	97.92	105.58	111.98	116.92	119.58	121.19
45	2.00	78.97	88.99	97.28	104.88	111.22	116.10	118.72	120.31
46	2.00	78.36	88.40	96.65	104.19	110.47	115.29	117.87	119.45
47	2.00	77.78	87.82	96.04	103.52	109.72	114.49	117.04	118.59
48	2.00	77.21	87.25	95.43	102.85	108.99	113.70	116.22	117.75
49	2.00	76.66	86.69	94.83	102.18	108.26	112.91	115.40	116.91
50	2.00	76.12	86.14	94.23	101.52	107.54	112.14	114.59	116.09
51	2.00	75.58	85.59	93.63	100.87	106.82	111.37	113.79	115.27
52	2.00	75.05	85.03	93.03	100.21	106.10	110.60	113.00	114.45
53	2.00	74.51	84.48	92.43	99.55	105.39	109.83	112.20	113.64
54	2.00	73.99	83.93	91.84	98.90	104.68	109.08	111.42	112.84
55	2.00	73.46	83.38	91.25	98.25	103.98	108.33	110.65	112.05
56	2.00	72.94	82.84	90.66	97.61	103.28	107.58	109.88	111.27
57	2.00	72.42	82.29	90.07	96.96	102.58	106.85	109.12	110.49
58	2.00	71.90	81.74	89.47	96.32	101.89	106.11	108.36	109.72
59	2.00	71.36	81.18	88.87	95.66	101.19	105.37	107.60	108.95
60	2.00	70.82	80.62	88.26	95.00	100.48	104.63	106.83	108.17
61	2.00	70.26	80.03	87.64	94.33	99.77	103.87	106.06	107.38

62	2.00	69.70	79.44	87.01	93.65	99.04	103.11	105.28	106.59
63	2.00	69.12	78.83	86.36	92.96	98.31	102.34	104.49	105.79
64	2.00	68.53	78.22	85.70	92.26	97.56	101.56	103.69	104.98
65	2.00	67.93	77.59	85.03	91.54	96.80	100.77	102.88	104.16
66	2.00	67.32	76.94	84.34	90.81	96.03	99.97	102.06	103.33
67	2.00	66.70	76.29	83.65	90.07	95.25	99.15	101.23	102.48
68	2.00	66.08	75.63	82.95	89.32	94.46	98.33	100.39	101.63
69	2.00	65.46	74.97	82.23	88.56	93.66	97.50	99.54	100.78
70	2.00	64.84	74.30	81.52	87.80	92.85	96.66	98.69	99.91
71	2.00	64.21	73.62	80.79	87.02	92.04	95.82	97.83	99.04
72	2.00	63.58	72.94	80.06	86.24	91.22	94.97	96.96	98.16
73	2.00	62.94	72.24	79.31	85.45	90.39	94.10	96.08	97.27
74	2.00	62.30	71.54	78.56	84.65	89.55	93.23	95.19	96.38
75	2.00	61.66	70.84	77.80	83.84	88.70	92.36	94.30	95.47
76	2.00	61.01	70.13	77.04	83.03	87.84	91.47	93.40	94.56
77	2.00	60.37	69.42	76.27	82.21	86.98	90.58	92.49	93.65
78	2.00	59.74	68.71	75.50	81.39	86.12	89.69	91.58	92.73
79	2.00	59.11	68.00	74.73	80.56	85.25	88.79	90.67	91.81
80	2.00	58.49	67.29	73.96	79.73	84.38	87.89	89.75	90.88
