# Supplementary material for: Estimation of age and sex-specific Glomerular Filtration Rate and its association with mortality and atherosclerotic cardiovascular outcomes in the Abu Dhabi population; A Retrospective Cohort Study
Source: J Nephrol. 2025 Aug 5;38(7):1957–67. doi: 10.1007/s40620-025-02347-w (PMC12484320; doi:10.1007/s40620-025-02347-w)
Supplement: Supplementary file 2 — Supplementary file2 (DOCX 15 KB) [file 40620_2025_2347_MOESM2_ESM.docx]

Appendix 2 Subjects’ characteristics

| ADRS 2011-2013 cohort | | |
| --- | --- | --- |
|  | Mean | SD |
| BMI | 28.8 | 6.3 |
| GFR | 111.3 | 18.6 |
| SBP | 120.5 | 15.2 |
| DBP | 73.5 | 10.5 |
| Total cholesterol | 4.8 | 2.1 |
| HBA1C | 5.9 | 1.1 |
| HDL | 1.3 | 0.3 |
| Age | 38.9 | 15 |
| Prevelance of Chronic Diseases | | |
| Diabetes Mellitus | 22.2% | |
| Hypertension | 19.3% | |
| Smoking among males | 16.30% | |
