# Supplementary figures and images for: Estimation of age and sex-specific Glomerular Filtration Rate and its association with mortality and atherosclerotic cardiovascular outcomes in the Abu Dhabi population; A Retrospective Cohort Study
Source: J Nephrol. 2025 Aug 5;38(7):1957–67. doi: 10.1007/s40620-025-02347-w (PMC12484320; doi:10.1007/s40620-025-02347-w)

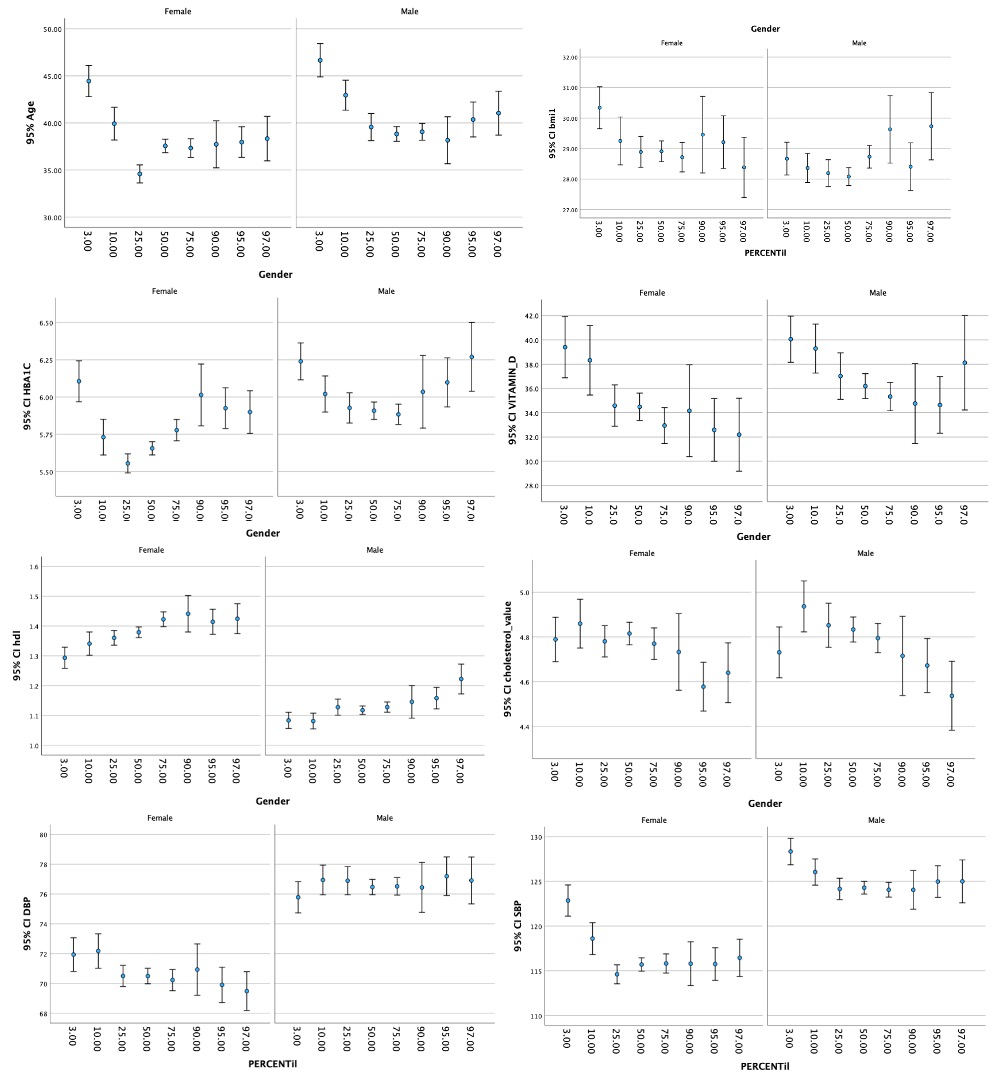

Supplement: Supplementary file 3 — Supplementary file3 (JPG 160 KB) [file 40620_2025_2347_MOESM3_ESM.jpg]
